# The Role of Early Intubation in Status Epilepticus with Out-of-Hospital Onset: A Large Prospective Observational Study

**DOI:** 10.3390/jcm13040936

**Published:** 2024-02-06

**Authors:** Gianni Turcato, Giada Giovannini, Simona Lattanzi, Niccolò Orlandi, Giulia Turchi, Arian Zaboli, Francesco Brigo, Stefano Meletti

**Affiliations:** 1 Department of Internal Medicine, Hospital of Santorso (AULSS-7), 36014 Santorso, Italy; gianni.turcato@aulss7.veneto.it; 2 Neurology Department, Azienda Ospedaliera-Universitaria di Modena, 41124 Modena, Italy; giovannini.giada@gmail.com (G.G.); giu.turchi@gmail.com (G.T.); meletti.stefano@unimore.it (S.M.); 3 PhD Programm in Clinical and Experimental Medicine, University of Modena and Reggio-Emilia, 41121 Modena, Italy; 4 Neurological Clinic, Department of Experimental and Clinical Medicine, Marche Polytechnic University, 60121 Ancona, Italy; alfierelattanzisimona@gmail.com; 5 Department of Biomedical, Metabolic, and Neural Sciences, University of Modena and Reggio-Emilia, 41121 Modena, Italy; 6 Innovation, Research and Teaching Service (SABES-ASDAA), Teaching Hospital of the Paracelsus Medical Private University (PMU), 39010 Bolzano, Italy

**Keywords:** status epilepticus, intubation, outcomes

## Abstract

**Background:** this study aimed to evaluate the role of early airway management and intubation in status epilepticus (SE) with out-of-hospital onset. **Methods:** We included all patients with out-of-hospital SE onset referred to the emergency department of the Academic Hospital of Modena between 2013 and 2021. Patients were compared according to out-of-hospital airway management (intubation versus non-intubation) and a propensity score was performed for clinical variables unevenly distributed between the two groups. **Results:** We evaluated 711 patients with SE. A total of 397 patients with out-of-hospital SE onset were eventually included; of these, 20.4% (81/397) were intubated before arrival at the hospital. No difference was found in the clinical characteristics of patients after propensity score matching. The 30-day mortality in the propensity group was 19.4% (14/72), and no difference was found between intubated (7/36, 19.4%) and non-intubated (7/36, 19.4%) patients. No difference was found in SE cessation. Compared to non-intubated patients, those who underwent out-of-hospital intubation had a higher risk of progression to refractory or super-refractory SE, greater worsening of mRS values between hospital discharge and admission, and lower probability of returning to baseline condition at 30 days after SE onset. **Conclusions:** Early intubation for out-of-hospital SE onset is not associated with improved patient survival even after balancing for possible confounders. Further studies should evaluate the timing of intubation and its association with first-line treatments for SE and their efficacy. In addition, they should focus on the settings and the exact reasons leading to intubation to better inform early management of SE with out-of-hospital onset.

## 1. Introduction

Status epilepticus (SE) is a time-dependent medical and neurological emergency associated with high risk of long-term consequences and mortality, requiring a prompt diagnosis and timely therapeutic management [1,2,3,4]. The annual incidence rates of this life-threatening condition range from 1.29 to 73.7/100,000 adults (95% confidence interval, CI: 76.6–80.3) [5].

The impairment of consciousness, which often occurs at SE onset (particularly if generalized convulsive or non-convulsive in coma), can require early airway protection with intubation by an emergency physician. The use of an adequate dose of benzodiazepines given as first-line treatment for SE, but also SE itself, could cause respiratory insufficiency, cardiac dysrhythmia, or blood pressure instability, making intubation mandatory [2,3]. However, sometimes patients with SE undergo intubation in the absence of any of the aforementioned reasons. 

Although such an airway management is performed and, in some instances, recommended [6,7,8], no study has investigated the role of early intubation in the management of SE with out-of-hospital onset [9,10]. Conversely, although the importance of ensuring the patency of the airways is widely recognized, especially during transport to the hospital, unnecessary intubation can be associated with risks. Thus, excessively aggressive airway management might not necessarily represent the best option for treating SE at onset, particularly if SE is not associated with severe respiratory changes, suggesting that alternative approaches could be considered. There remains a need to further clarify the role of intubation in SE with out-of-hospital onset.

This study aimed to evaluate the relationship between early intubation and short-term outcomes in patients with SE with out-of-hospital onset.

## 2. Methods

### 2.1. Study Design, Setting, and Patients 

We conducted a retrospective analysis of prospective data collected at the Emergency Department (ED) of the Civil Hospital of Baggiovara (Modena, Italy). All patients aged 14 years of age or older and with a diagnosis of SE from 1 September 2013 to 31 October 2021 were consecutively recruited; we included only patients with SE with out-of-hospital onset. 

Patients with out-of-hospital emergencies, including SE, are generally cared for at the scene and in the ambulance by physicians and emergency nurses. All out-of-hospital providers have access to rapid sequence intubation with induction agents and paralytics, and after intubation, all patients are immediately transported to the hospital.

Prior to 2015, SE was defined as an epileptic seizure lasting 5 min or more or as two or more distinct seizures with no complete recovery of consciousness between them [11]. After 2015, the definition of the International League Against Epilepsy (ILAE) was systematically adopted and prospectively applied [1]. Consequently, the operative time indicating when a seizure is likely to be prolonged and leads to continuous seizure activity (i.e., SE) was set at 5 min for tonic–clonic SE, 10 min for focal SE with impaired consciousness, and 10–15 min for absence SE. All cases of SE occurring before 2015 were reviewed by two study authors (MS and GG) to ensure that all met the ILAE diagnostic criteria. Cases of non-convulsive SE were diagnosed according to the Salzburg criteria [12,13]. Patients who underwent intubation later in their hospital course (e.g., in the emergency department in the intensive care unit) were excluded.

A prespecified and standard dataset was used to prospectively collect demographic and clinical information, including age; sex; setting of SE onset (out-of-hospital or in-hospital); Glasgow Coma Scale at SE onset; impairment of consciousness before treatment (i.e., stupor or coma); etiological ILAE classification [1], in which acute symptomatic causes were divided into hypoxic or non-hypoxic; SE semiology according to ILAE classification [1]; prior history of epilepsy; medical history and comorbidities; abnormal electroencephalogram (EEG) (lateralized periodic discharges, after status ictal discharges, generalized sharply and/or triphasic period potentials, and spontaneous burst suppression) [14]; and scores of EMSE and STESS [14,15]. The form was filled in by the first physician (neurologist or neurointensivist) taking care of the patient. 

Treatment followed an internal protocol (publicly available at https://salute.regione.emilia-romagna.it/epilessia/PDTASE_AOU.pdf, accessed on 15 December 2021; Supplementary Materials) based on the recommendations of international guidelines [6,7,8].

### 2.2. Outcome

The primary outcome of this study was 30-day mortality. Secondary outcomes were as follows.

1. Progression to refractory or super-refractory SE (the latter defined as a SE “that continues or recurs 24 h or more after the onset of anaesthetic therapy, including those cases where SE recurs on the reduction or withdrawal of anaesthesia” [16]); 2. worsening of modified Rankin Scale (mRS) at discharge compared to admission; 3. SE cessation defined according to the Sustained Effort Network for treatment of Status Epilepticus (SENSE) study as follows: cessation of SE within the first hour after treatment initiation for generalized convulsive SE and within 12 h after treatment initiation for other SE types; and 4. lack of return to baseline condition at 30 days [17].

Data on the follow-up of patients, including SE cessation and their mortality, as well as mRS at admission and at discharge, were obtained from the SE dataset used to prospectively collect information and were confirmed through the registry office.

### 2.3. Ethical Aspects

This study was approved by the local ethics committee (ethics committee approval number 556/2018/OSS/AOUMO-RF-2016-02361365) and was conducted according to the ethical principles for medical research involving human subjects according to the Declaration of Helsinki.

### 2.4. Statistical Analysis

Continuous variables were reported as median and interquartile range (IQR) or as mean and standard deviation (SD), depending on the underlying distribution. Categorical variables were reported as percentage and number of events out of the total. Univariate comparisons were performed with the Mann–Whitney test, *t*-test, Fischer Exact test, or Chi-square test, whichever was appropriate. 

We investigated the possible independent association between intubation for SE with out-of-hospital onset and 30-day mortality using a multivariate model with Cox regression adjusted for the variables found to be significant in the univariate analyses and included as possible multivariate confounders. A logistic regression with the backwise regression method was performed and the risk ratios (RRs) with the respective 95% confidence intervals (CIs) were reported. Survival analysis between the two groups (intubated versus non-intubated) was conducted with the Kaplan–Meier method using the log-rank test for comparisons.

Subsequently, considering the different baseline conditions that could have influenced the outcome and the fact that the study did not include pre-enrollment selection criteria, we included the baseline variables that were unbalanced between the two treatment groups into a propensity score matching. 

After propensity score matching, we repeated the analyses to evaluate the independent association between the intubation and study outcomes. 

All tests were two-sided and a *p*-value of 0.050 was considered statistically significant. Statistical analyses were performed with Stata^®^ version 16.0 (StataCorp, College Station, TX, USA).

## 3. Data Availability

Upon request from qualified investigators, we will share the anonymized data.

### 3.1. Results

Out of the 711 patients evaluated for SE, 397 patients with out-of-hospital SE onset were eventually included. Of these, 20.4% (81/397) were intubated before arrival at the hospital. Patients who responded to first-line (benzodiazepine) or second-line (intravenous medication) treatments included 229/397 (57.7%). Responders were 13/81 (16.0%) intubated and 216/316 (68.4%) non-intubated patients. The baseline clinical characteristics of patients are reported in Table 1.

Compared to non-intubated patients, those who had been intubated were younger, were more often male, had lower Glasgow Coma Scale values, had higher prevalence of stupor/coma, had acute symptomatic hypoxic SE etiology, presented with generalized convulsive and myoclonic SE, and had ischemic heart disease as a comorbid condition. Conversely, patients who did not undergo intubation had a higher prevalence of acute symptomatic non-hypoxic and remote symptomatic SE etiology, non-convulsive SE, and dementia as comorbidity. SE cases due to toxic etiologies, especially overdoses and toxic ingestion, were not represented in this patient cohort. 

### 3.2. 30-Day Mortality and Secondary Outcomes

Patients who died within 30 days from SE onset were 18.9% (75/397). Among patients with out-of-hospital intubation, 30-day mortality was 28.4% (23/81), whereas in non-intubated patients, it was 16.5% (52/316) (*p* = 0.017).

The clinical characteristics associated with the risk of 30-day mortality are reported in Table 2. Patients who died within 30 days from SE onset underwent more often out-of-hospital intubation, were younger, and had lower Glasgow Coma Scale values, had a higher prevalence of stupor/coma, acute symptomatic hypoxic SE etiology, no history of epilepsy, specific comorbidities (ischemic heart disease, cerebrovascular disease, dementia, tumor, chronic obstructive pulmonary disease, liver failure, and chronic kidney disease), malignant EEG patterns, and higher EMSE and STESS values. Conversely, patients who survived at 30 days after SE onset had a higher prevalence of remote symptomatic SE etiology. 

In the Cox multivariate analysis, performed with the variables found to be significant at the previous univariate analysis, out-of-hospital intubation was not a risk factor for 30-day mortality. Factors associated with increased risk of 30-day mortality were age, lower values in Glasgow Coma Scale at SE onset, ischemic heart disease, cerebrovascular disease, chronic obstructive pulmonary disease, and higher EMSE values (Table 3).

**Figure 1:**
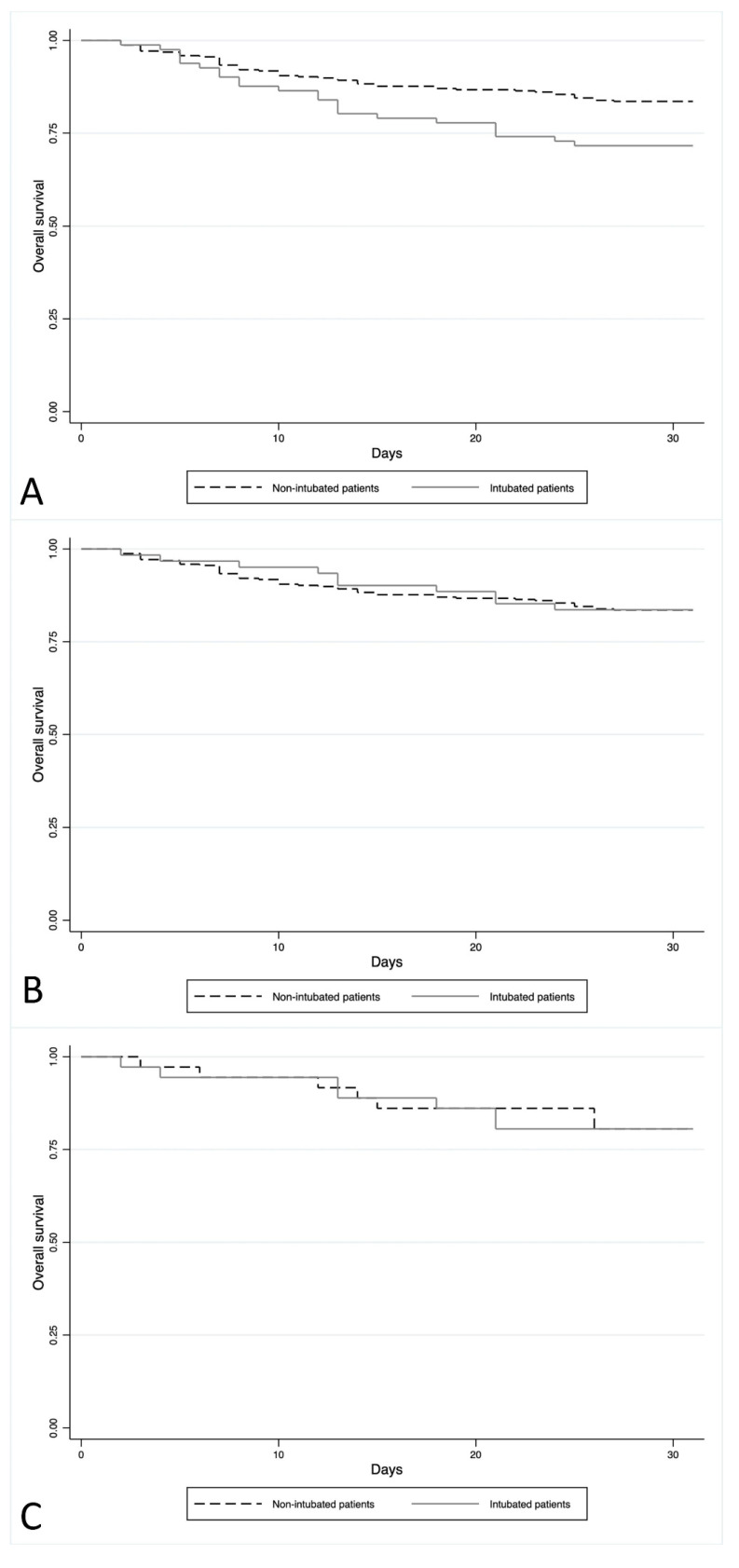
(**A**) The Kaplan–Meier analysis of all patients. Outcome: 30-day survival of patients with out-of-hospital intubation compared to non-intubated patients. (**B**) The Kaplan–Meier analysis of patients without acute hypoxic SE. Outcome: 30-day survival of patients with out-of-hospital intubation compared to non-intubated patients. (**C**) The Kaplan–Meier analysis of the propensity groups. Outcome: 30-day survival of patients with out-of-hospital intubation compared to non-intubated patients.

The Kaplan–Meier analysis showed that the average survival of patients with intubation was 25.6 days compared to 27.8 days for patients who did not undergo out-of-hospital intubation (*p* = 0.014) (Figure 1A).

Differences between patients with and without out-of-hospital intubation were found also in secondary outcomes (Table 4). Compared to non-intubated patients, those who underwent intubation had a lower prevalence of in-hospital mortality, worsening of mRS values between hospital discharge and admission, and lack of return to baseline condition at 30 days. Furthermore, intubated patients had a higher prevalence of progression to refractory and super-refractory SE.

Excluding acute hypoxic patients (*n* = 20), there remained 16.2% (61/377) of patients with out-of-hospital intubation. After the exclusion of this specific etiology, no difference was found in the 30-day mortality between patients with out-of-hospital intubation (10/61, 16.4%) and those not undergoing intubation (52/316, 16.5%) (*p* > 0.05). The Kaplan–Meier analysis also showed no difference in the average survival between intubated (mean survival: 28.1 days) and non-intubated patients (mean survival: 27.8 days) (*p* = 0.960) (Figure 1B). 

### 3.3. Propensity Matching of Patients

A propensity score was performed with the clinical variables found to be differently distributed between intubated and non-intubated patients. A one-to-one statistical matching was carried out, obtaining a restricted subset of 72 patients equally distributed into 36 pairs of patients with and without intubation. No difference was found in the clinical characteristics of patients after propensity score matching. The 30-day mortality in the propensity group was 19.4% (14/72), and no difference was found between intubated (7/36, 19.4%) and non-intubated (7/36, 19.4%) patients (*p* > 0.05). 

The analysis of KM showed no difference in mortality between the two groups of patients, with a comparable average survival (27.8 days vs. 27.5 days; *p* = 0.986) (Figure 1C).

Other outcomes analyzed in the propensity group are reported in Table 5. No difference was found in SE cessation. Compared to non-intubated patients, those who underwent out-of-hospital intubation had a higher risk of progression to refractory or super-refractory SE, greater worsening of mRS values between hospital discharge and admission, and lower probability of returning to baseline condition at 30 days after SE onset.

## 4. Discussion

Our study, conducted in a large prospective cohort of patients with SE with out-of-hospital onset, evaluated whether there is an association between early intubation and short-term outcomes, including mortality and progression to refractory and super-refractory SE. 

In the acute setting, ensuring the patency of airways is always a priority, and intubation represents a life-saving procedure that cannot be postponed if the patient is unable to maintain adequate respiratory activity. In clinical practice, early intubation is a consolidated practice, although not always supported by robust evidence, particularly for managing acute conditions without severe cardiovascular or respiratory instability [18,19]. 

In the present study, 20.4% of patients (81/397) were intubated before arrival at the hospital. This high frequency of early intubation for SE with out-of-hospital onset, having not yet progressed to refractory SE, can be partly explained by the geographical peculiarities of our study. Our hospital serves a very large territory, and it is likely that sometimes, the large distances from the setting where the SE occurred to the emergency department prompted some emergency physicians to opt for early intubation to ensure a safe transport to the hospital rather than for specific clinical reasons.

In our study, patients undergoing out-of-hospital intubation were younger and had lower Glasgow Coma Scale values at SE onset, a higher prevalence of acute symptomatic SE with hypoxic etiology, generalized seizure SE, and ischemic heart disease as a comorbidity. In addition, a lower prevalence of patients who responded to either first-line (benzodiazepine) or second-line (intravenous medication) treatments were found among those non-intubated. These findings reflect confounding by indication, as patients with more severe conditions (including hypoxemia as the cause of SE) were more likely to need earlier intubation. Patients with more severe SE are less likely to respond to first-line treatment and are more likely to respond to second-line medications or anesthetics.

These findings are consistent with the recent results of a post hoc analysis of the SENSE registry, an observational cohort study which prospectively recruited all consenting adults with SE from nine centers in three German-speaking countries. Although it was performed in patients with refractory SE continuing despite treatment with two non-sedative antiseizure medications and was not restricted to SE cases with out-of-hospital onset, this study showed that intubation was associated with younger age, more severe consciousness impairment, more severe SE types (generalized tonic–clonic or non-convulsive with coma), higher severity score (STESS), and acute etiologies [10]. Of note, factors that in this study were associated with the decision of intubating refractory patients were mostly similar to those that we found affecting the choice of intubation in the earliest stage of SE before its progression to refractory SE.

Overall, our data indicate that severe cardiopulmonary impairment leading to acute hypoxic SE or airway obstruction (in the context of impaired consciousness, such as during generalized tonic–clonic SE or non-convulsive SE with coma) are associated with out-of-hospital intubation. Furthermore, an impairment in the degree of consciousness, albeit transient and not necessarily affecting respiratory functions, could also lead to early intubation. However, our study shows that performing an intubation in non-respiratory conditions is not associated with an improved outcome, suggesting that—at least in selected cases—a more conservative approach should be considered and justified.

In the analysis of this large prospective cohort of SE patients, before performing any propensity score matching, intubated patients had a higher risk of 30-day mortality. After performing a propensity score matching to balance the clinical variables unevenly distributed between intubated and non-intubated patients, no difference was found in 30-day mortality, hospital mortality, or SE cessation between the two groups. This suggests that out-of-hospital intubation is not necessarily associated with better short-term outcomes of SE patients. Moreover, patients without out-of-hospital intubation had a lower probability of returning to baseline condition at 30 days after SE onset, with significant worsening of mRS between hospital discharge and admission. 

Ensuring an effective airway patency is widely recommended for the treatment of many acute conditions and it is warranted for the management of generalized convulsive SE. However, intubation is a procedure that carries the risk of complications and requires a subsequent hospital admission and high intensity of care. Appraising the appropriateness and the clinical effectiveness of this procedure appears important, considering the clinical and organizational consequences it entails. Status epilepticus without a concomitant cardiovascular or respiratory impairment or SE associated only with mild and fluctuating impairment of consciousness could be conditions where the risks of an indiscriminate early intubation outweigh its benefits.

The positive association between early intubation and mortality in the general cohort could be explained by an apparent higher intrinsic severity of SE and the inclusion of hypoxic SE (in this case, intubation would represent a confounder by indication). However, the presence of impaired consciousness might not justify an early intubation unless associated with a concomitant cardiovascular instability or respiratory impairment. As the results of the propensity matching clearly show, after balancing for the baseline characteristics and severity of SE, early intubation does not increase the chances of survival. Undoubtedly, intubation must be considered immediately and systematically in the first evaluation of every patient with out-of-hospital SE onset. However, the rapid assessment of SE severity and its response to first-line medications could avoid an indiscriminate and unnecessary use of intubation.

Our study has some limitations. No sample size calculations were performed, but we adopted a convenience sample. However, due to the relatively low incidence of SE and the peculiarities of the setting (out-of-hospital onset), the study sample was quite large. The relatively small number of patients in the matched cohort limits the generalizability of propensity score analysis. Details on the setting and precise reasons for intubation (e.g., airway protection, respiratory insufficiency, cardiac dysrhythmia, blood pressure instability, or SE treatment) were not recorded, preventing us from analyzing which variables influenced the clinical decisions for this procedure. The decision to intubate the patients was left to the treating physician and was not based on a predefined protocol. Although a protocol for the treatment of SE was adopted, it is possible that some patients received inappropriate doses of benzodiazepines or of second-line medications, which could have resulted in a greater likelihood of intubation and poorer outcomes. The lack of further details on correct dosing, reflecting the real-world setting where this study was conducted, prevented us from assessing its impact on patient outcomes. Although the timing related to the administration of a benzodiazepine and intubation could also have affected the outcome, as delays in administering appropriate treatment might have led to worse prognosis, we did not have enough information to evaluate the potential impact of this factor. Similarly, we could not provide details on the number of patients with benzodiazepine-resistant SE who responded to a second-line medication.

This study took place over nine years, with the risk of secular trends possibly altering airway management among the physicians. However, it is unlikely that major changes to procedures, drugs, devices, and the rate of intubation have occurred over the course of the study, as no specific guidelines on airway management have been published in this timeframe. The timing of intubation, the use of non-invasive methods to support the airway, including supplemental oxygen, and details on the administration of first-line treatments and their efficacy were not available. When planning this study, we were aware of the risk of confounding by indication, especially hypoxemia, for intubation, expecting that patients with more severe conditions and worse outcomes were more likely to need earlier intubation. Unfortunately, confounding by indication is unavoidable in observational studies and can be adequately addressed only by adopting an experimental design, which is clearly unfeasible in this context. Hence, to mitigate the potential risk of confounding due to treatment selection, we performed a propensity matched analysis by balancing clinical and prognostic characteristics of groups at baseline. This allowed us to explore causal relationships using observational data with a robust methodology. The lack of difference in some outcomes in the propensity cohort could be a function of the limited sample size, emphasizing the need for further studies in this field. Finally, we evaluated the functional outcome using the mRS, which requires assessment of the return to daily activities and therefore can only be speculatively determined at hospital discharge. 

## 5. Conclusions

This study has provided some preliminary evidence that early intubation for SE with out-of-hospital onset in an acute setting is not necessarily associated with improved patient survival even after balancing for possible confounders. Association is not causation, and our findings do not imply any causal relationship between early intubation and lack of a consistent beneficial effect on outcomes. However, although limited by the relatively small sample size and residual confounding, the results of our study are worth being carefully considered and further explored. 

Particularly if not adequately standardized, early intubation could carry risks for the patient, especially when performed prematurely, before arrival at the hospital, or in patients who do not necessarily require it. Furthermore, a premature intubation prevents the neurologist from adequately evaluating the neurological status of patients. Conservative management is appropriate for many patients with SE without an additional comorbidity and negative impact on prognosis.

Future studies evaluating the timing of intubation and its association with first-line treatments and their efficacy, as well as the settings and the exact reasons leading to intubation, could provide further indications about the individualized risks and benefits associated with this procedure for early management of SE with out-of-hospital onset. They should clarify whether the lack of a consistent association between early intubation for out-of-hospital SE and better outcomes is true or spurious. Currently, the optimal timing and criteria for intubation remain to be fully elucidated, preferably through high-quality, adequately powered randomized trials.

## Figures and Tables

**Table 1:** Baseline clinical characteristics of patients.

Variable	All Patients	Non-Intubated Patients	Intubated Patients	*p*-Value
Patients, *n* (%)	322 (100)	316 (79.6)	81 (20.4)	
Age, years, mean (SD)		68.7 (18.2)	62 (16.4)	0.003
Sex, *n* (%)				0.023
Male	169 (42.6)	124 (39.6)	44 (54.3)	
Female	228 (57.4)	191 (60.4)	37 (45.7)	
GCS at SE onset, mean (SD)	11.1 (3.9)	12.1 (3.1)	6.8 (4.1)	<0.001
Impaired consciousness before treatment, *n* (%)	82 (20.7)	31 (9.8)	51 (63)	<0.001
Etiological classification, *n* (%)				
Acute symptomatic, hypoxic	20 (5.0)	0 (0)	20 (24.7)	<0.001
Acute symptomatic, non-hypoxic	181 (45.6)	152 (48.1)	29 (35.8)	0.031
Remote symptomatic	87 (21.9)	77 (24.4)	10 (12.3)	0.023
Progressive symptomatic	86 (21.7)	70 (22.2)	16 (19.8)	0.763
Other	23 (5.8)	17 (5.4)	6 (7.4)	0.430
SE semiology, *n* (%)				
Generalized convulsive	90 (22.7)	60 (19)	30 (37)	0.001
Focal motor	108 (27.2)	89 (28.2)	19 (23.5)	0.484
Non-convulsive	189 (47.6)	162 (51.3)	27 (33.3)	0.004
Myoclonic	10 (2.5)	5 (1.6)	5 (6.2)	0.034
Prior history of epilepsy, *n* (%)	166 (41.8)	139 (44)	27 (33.3)	0.101
Comorbidities, *n* (%)				
Ischemic heart disease	40 (10.1)	25 (7.9)	15 (18.5)	0.011
Cerebrovascular disease	45 (11.3)	40 (12.7)	5 (6.2)	0.117
Connective tissue disease	9 (2.3)	8 (2.5)	1 (1.2)	0.693
Diabetes mellitus	62 (15.6)	53 (16.8)	9 (11.1)	0.234
Heart Failure	18 (4.5)	16 (5.1)	2 (2.5)	0.548
Dementia	77 (19.4)	70 (22.2)	7 (8.6)	0.005
Ulcer	18 (4.5)	12 (3.8)	6 (7.4)	0.225
Hemiplegia	35 (8.8)	30 (9.5)	5 (6.2)	0.509
Tumor	48 (12.1)	37 (11.7)	11 (13.6)	0.702
Peripheral vascular disease	8 (2.0)	8 (2.5)	0 (0)	0.168
COPD	39 (9.8)	31 (9.8)	8 (9.8)	1.000
Liver failure	17 (4.3)	14 (4.4)	3 (3.7)	1.000
Chronic kidney disease	29 (7.3)	24 (7.6)	5 (6.2)	0.813
Abnormal EEG, *n* (%) *	126 (31.7)	85 (26.9)	41 (50.6)	<0.001
Prognostic scores				
EMSE	48.7 (36.1)	43.7 (30.8)	68.1 (47.2)	<0.001
STESS	2.7 (1.6)	2.4 (1.4)	3.7 (1.7)	<0.001

Legend: COPD: chronic obstructive pulmonary disease; EMSE: epidemiology-based mortality score in status epilepticus; GCS: Glasgow Coma Scale; SD: standard deviation; SE: status epilepticus; STESS: status epilepticus severity score. * malignant EEG pattern as defined by the EMSE score.

**Table 2:** Clinical characteristics associated with the risk of 30-day mortality.

Variable	Survivors	Non-Survivors	*p*-Value
Patients, *n* (%)	322 (81.1)	75 (18.9)	
Out-of-hospital intubation			0.017
No	264 (82)	52 (69.3)	
Yes	58 (18)	23 (30.7)	
Age, years, mean (SD)	64.6 (18.2)	79.4 (11.3)	<0.001
Sex, *n* (%)			0.121
Male	131 (40.7)	38 (50.7)	
Female	191 (59.3)	37 (49.3)	
GCS at SE onset, mean (SD)	11.6 (3.6)	8.7 (4.1)	<0.001
Impaired consciousness before treatment, *n* (%)	53 (16.5)	29 (38.7)	<0.001
Etiological classification, *n* (%)			
Acute symptomatic, hypoxic	7 (2.2)	13 (17.3)	<0.001
Acute symptomatic, non-hypoxic	143 (44.4)	58 (50.7)	0.364
Remote symptomatic	80 (24.8)	7 (9.3)	0.003
Progressive symptomatic	75 (23.3)	11 (14.7)	0.120
Other	17 (5.3)	6 (8.0)	0.408
SE semiology, *n* (%)			
Generalized convulsive	75 (23.3)	15 (20)	0.646
Focal motor	88 (27.3)	20 (26.7)	1.000
Non-convulsive	153 (47.5)	36 (48)	1.000
Myoclonic	6 (1.9)	4 (5.3)	0.099
Prior history of epilepsy, *n* (%)	145 (45)	21 (28)	0.009
Comorbidities, *n* (%)			
Ischemic heart disease	23 (7.1)	17 (22.7)	<0.001
Cerebrovascular disease	25 (7.8)	20 (26.7)	<0.001
Connective tissue disease	9 (2.8)	0 (0)	0.218
Diabetes mellitus	49 (15.2)	13 (17.3)	0.724
Heart Failure	12 (3.7)	6 (8)	0.123
Dementia	50 (15.5)	27 (36)	<0.001
Ulcer	11 (3.4)	7 (9.3)	0.056
Hemiplegia	27 (8.4)	8 (10.7)	0.503
Tumor	30 (9.3)	18 (24)	0.001
Peripheral vascular disease	5 (1.6)	3 (4)	0.177
COPD	25 (7.8)	14 (18.7)	0.008
Liver failure	9 (2.8)	8 (10.7)	0.006
Chronic kidney disease	4 (1.2)	5 (6.7)	0.014
Abnormal EEG, *n* (%) *	79 (24.5)	47 (62.7)	<0.001
Prognostic scores			
EMSE	40.4 (30.2)	84.3 (37.7)	<0.001
STESS	2.4 (1.5)	3.7 (1.6)	<0.001

Legend: COPD: chronic obstructive pulmonary disease; EMSE: epidemiology-based mortality score in status epilepticus; GCS: Glasgow Coma Scale; SD: standard deviation; SE: status epilepticus; STESS: status epilepticus severity score. * malignant EEG pattern as defined by the EMSE score.

**Table 3:** Factors associated with increased risk of 30-day mortality in the COX multivariate analysis.

Variable	Coefficient	Error	HR	95% Confidence Intervals	*p*-Value
Age	0.072	0.012	1.075	1.049–1.101	<0.001
GCS at SE onset	−0.075	0.031	0.928	0.872–0.987	0.017
Ischemic heart disease	0.646	0.290	1.907	1.080–3.368	0.026
Cerebrovascular disease	0.580	0.272	1.786	1.048–3.045	0.033
COPD	0.594	0.300	1.811	1.006–3.259	0.048
EMSE	0.020	0.003	1.020	1.014–1.026	<0.001

Legend: COPD: chronic obstructive pulmonary disease; EMSE: epidemiology-based mortality score in status epilepticus; GCS: Glasgow Coma Scale; SE: status epilepticus.

**Table 4:** Differences between patients with and without out-of-hospital intubation in secondary outcomes.

Outcome, *n* (%)	Non-Intubated Patients	Intubated Patients	*p*-Value
In-hospital mortality	47 (63.5)	27 (36.5)	<0.001
Progression to refractory or super-refractory SE	24 (27.9)	62 (72.1)	<0.001
Worsening of mRS between hospital discharge and admission	126 (70)	54 (30)	<0.001
SE cessation ^a^	110 (78)	31 (22)	0.603
Lack of return to baseline condition at 30 days	130 (71)	53 (29)	<0.001

Legend: ILAE: International League Against Epilepsy; mRS: modified Ranking Scale; SE: status epilepticus. ^a^ SE cessation was defined according to the Sustained Effort Network for treatment of Status Epilepticus (SENSE) study as follows: cessation of SE within the first hour after treatment initiation for generalized convulsive SE; cessation of SE within 12 h after treatment initiation for other SE types.

**Table 5:** Differences between patients with and without out-of-hospital intubation in secondary outcomes after propensity score matching.

Outcome, *n* (%)	Non-Intubated Patients	Intubated Patients	*p*-Value
In-hospital mortality	6 (46.2)	7 (53.8)	1.000
Progression to refractory or super-refractory SE	2 (7.1)	26 (92.9)	<0.001
Worsening of mRS between hospital discharge and admission	10 (30.3)	23 (69.7)	0.004
SE cessation ^a^	11 (50)	11 (50)	1.000
Lack of return to baseline condition at 30 days	10 (31.3)	22 (68.8)	0.004

Legend: ILAE: International League Against Epilepsy; mRS: modified Ranking Scale; SE: status epilepticus. ^a^ SE cessation was defined according to the Sustained Effort Network for treatment of Status Epilepticus (SENSE) study as follows: cessation of SE within the first hour after treatment initiation for generalized convulsive SE; cessation of SE within 12 h after treatment initiation for other SE types.

## Data Availability

The data presented in this study are available on request from the corresponding author.

## Supplementary Materials

The following supporting information can be downloaded at: https://www.mdpi.com/article/10.3390/jcm13040936/s1, Supplementary File S1: Internal Protocol for the Pharmacological Treatment of Status Epilepticus.
